# Improved G-AgarTrap: A highly efficient transformation method for intact gemmalings of the liverwort *Marchantia polymorpha*

**DOI:** 10.1038/s41598-018-28947-0

**Published:** 2018-07-17

**Authors:** Shoko Tsuboyama, Satoko Nonaka, Hiroshi Ezura, Yutaka Kodama

**Affiliations:** 1 Center for Bioscience Research and Education, Utsunomiya University, Tochigi, 321-8505 Japan; 2 Tsukuba Plant Innovation Research Center, University of Tsukuba, Ibaraki, 305-8572 Japan

## Abstract

Liverworts are key species for studies of plant evolution, occupying a basal position among the land plants. *Marchantia polymorpha* has emerged as a highly studied model liverwort, and many relevant techniques, including genetic transformation, have been established for this species. *Agrobacterium*-mediated transformation is widely used in many plant species because of its low cost. Recently, we developed a simplified *Agrobacterium*-mediated method for transforming *M. polymorpha*, known as AgarTrap (agar-utilized transformation with pouring solutions). The AgarTrap procedure, which involves culturing the liverwort tissue in various solutions on a single solid medium, yields up to a hundred independent transformants. AgarTrap is a simple procedure, requiring minimal expertise, cost, and time. Here, we investigated four factors that influence AgarTrap transformation efficiency: (1) humidity, (2) surfactant in the transformation buffer, (3) *Agrobacterium* strain, and (4) light/dark condition. We adapted the AgarTrap protocol for transforming intact gemmalings, achieving an exceptionally high transformation efficiency of 97%. The improved AgarTrap method will enhance the molecular biological study of *M. polymorpha*. Furthermore, this method provides new possibilities for improving transformation techniques for a variety of plant species.

## Introduction

*Marchantia polymorpha* is a dioecious liverwort belonging to the bryophyte family, a sister group of all other land plants^1–3^. This species has therefore been extensively studied to enhance our understanding of land plant evolution, with research focusing on its taxonomy, development, and physiology; furthermore, its nuclear, chloroplast, and mitochondrial genomes have all been sequenced^1,4–7^. However, Bowman *et al*. 2017^1^ suggested that the chloroplast and mitochondrial sequences reported previously^6,7^ were more similar to those of *Marchantia paleacea* than *M. polymorpha*. Nonetheless, the *M. polymorpha* research community has rapidly expanded recently, and various molecular biology techniques have been developed to study this key species, including particle bombardment- and *Agrobacterium*-mediated transformation, plastid transformation, homologous recombination-mediated gene targeting, and TALEN- and CRISPR/Cas9-mediated genome editing^8,9^.

*Agrobacterium*-mediated transformation is widely used for many plant species because it does not require any expensive equipment^10^. This technique involves three steps: (1) preparation of plant material, (2) co-culture of the material with *Agrobacterium tumefaciens* containing recombinant transfer DNA (T-DNA), and (3) antibiotic selection of transgenic cells. During the co-culture step, T-DNA is transferred from the *Agrobacterium* into the plant cell, where it is integrated into the genome to facilitate the expression of its constituent genes. Previous studies have determined that the co-culture conditions are the most important aspect affecting transformation efficiency, with the *Agrobacterium* strain used, duration of co-culture, *Agrobacterium* density, temperature, co-culture medium, and surfactants used having the greatest impact^11–15^.

**Figure 1:**
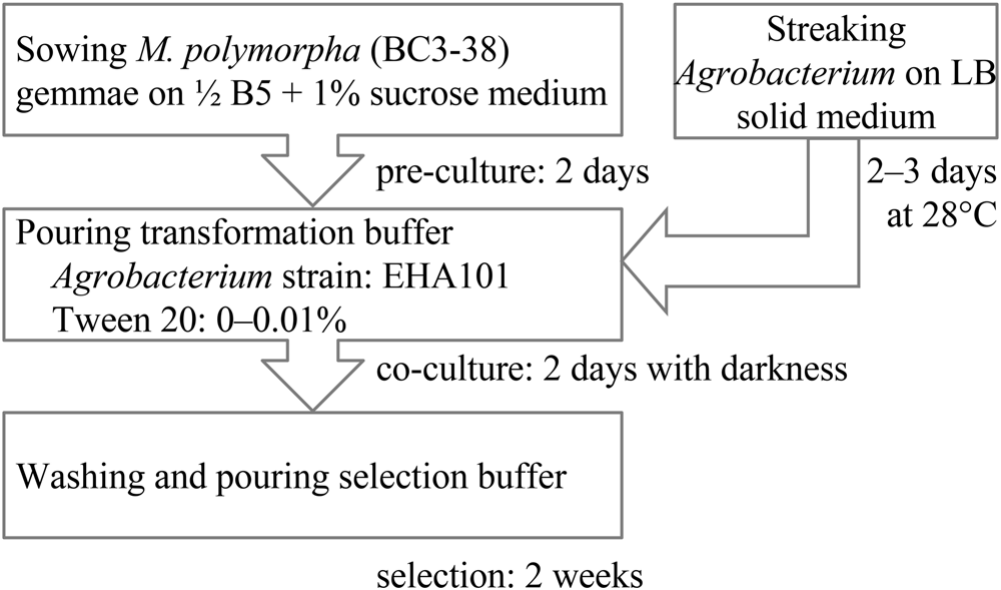
Flowchart of G-AgarTrap. Step I: Pre-culture of *M. polymorpha* gemmae/gemmalings on ½ B5 supplemented with 1% sucrose, and *Agrobacterium* on LB agar medium. Step II: Co-culture of *M. polymorpha* gemmalings with *Agrobacterium* on ½ B5 supplemented with 1% sucrose. Step III: Washing of *M. polymorpha* gemmalings and selection of transgenic cells on ½ B5 supplemented with 1% sucrose.

Recently, we developed a simplified *Agrobacterium*-mediated transformation method for *M. polymorpha*, which we named AgarTrap (agar-utilized transformation with pouring solutions)^16–18^. Like the general *Agrobacterium*-mediated transformation procedure, AgarTrap consists of three steps: (1) pre-culture of *M. polymorpha* tissue, (2) co-culture of the tissue with *Agrobacterium* containing recombinant T-DNA, and (3) selection of transgenic cells. A unique feature of AgarTrap is that none of these steps requires liquid medium culture; rather, the appropriate solutions are simply poured onto the solid medium in a single Petri dish (Fig. 1)^16–18^. We previously developed and optimized AgarTrap for use with *M. polymorpha* sporelings (S-AgarTrap), intact gemmae/gemmalings (G-AgarTrap), and pieces of mature thallus (T-AgarTrap), achieving transformation efficiencies of approximately 20% (F_1_ spores by crossing male Tak-1 with female Tak-2 strains), 60% (female BC3-38 strain), and 70% (female BC3-38 strain), respectively^16–18^. Despite its low transformation efficiency, S-AgarTrap results in numerous transformants, because spores are produced abundantly, rendering it suitable for the large-scale production of transformants (e.g., T-DNA insertion mutants)^16^. However, because spores are produced by sexual reproduction, S-AgarTrap transformants do not have a uniform genetic background. G-AgarTrap can be used to produce transformants in a genetically uniform background, because the gemmae develop from single cells asexually generated within the gemma cup on a mature thallus^17,19^. Similarly, T-AgarTrap results in transformants with uniform genetic backgrounds, because the cells are obtained from mature thalli^18^; however, fewer individual transformants were obtained using T-AgarTrap than G-AgarTrap despite their respective transformation efficiencies, because the pieces of mature thallus were larger than the gemmae and fewer could be included in a single Petri dish. Thus, of these three methods, G-AgarTrap appears to be the best choice for producing transgenic *M. polymorpha*; however, because the transformation efficiency of G-AgarTrap was relatively low (approximately 60% in the female BC3-38 strain when using ½ B5 medium supplemented with 1–2% sucrose), this approach needed improvement. As the co-culture step is the most critical for efficient transformation^11,15^, the transformation efficiency of G-AgarTrap would likely be improved by optimizing this step.

In our previous study, we optimized several factors of AgarTrap transformation, including the pre-culture period of *M. polymorpha* tissue, the duration of co-culture, *Agrobacterium* density (OD_600_ in transformation buffer), acetosyringone concentration in the transformation buffer, medium composition, and *Agrobacterium* culture conditions^16–18^. In the present study, we investigated four additional co-culture factors: (1) humidity, (2) surfactant in the transformation buffer, (3) *Agrobacterium* strain, and (4) light/dark condition. We also fine-tuned the pre-culture period, ultimately achieving an exceptionally high transformation efficiency for the G-AgarTrap procedure, of nearly 100%.

## Results

### Humidity conditions during co-culture

**Figure 2:**
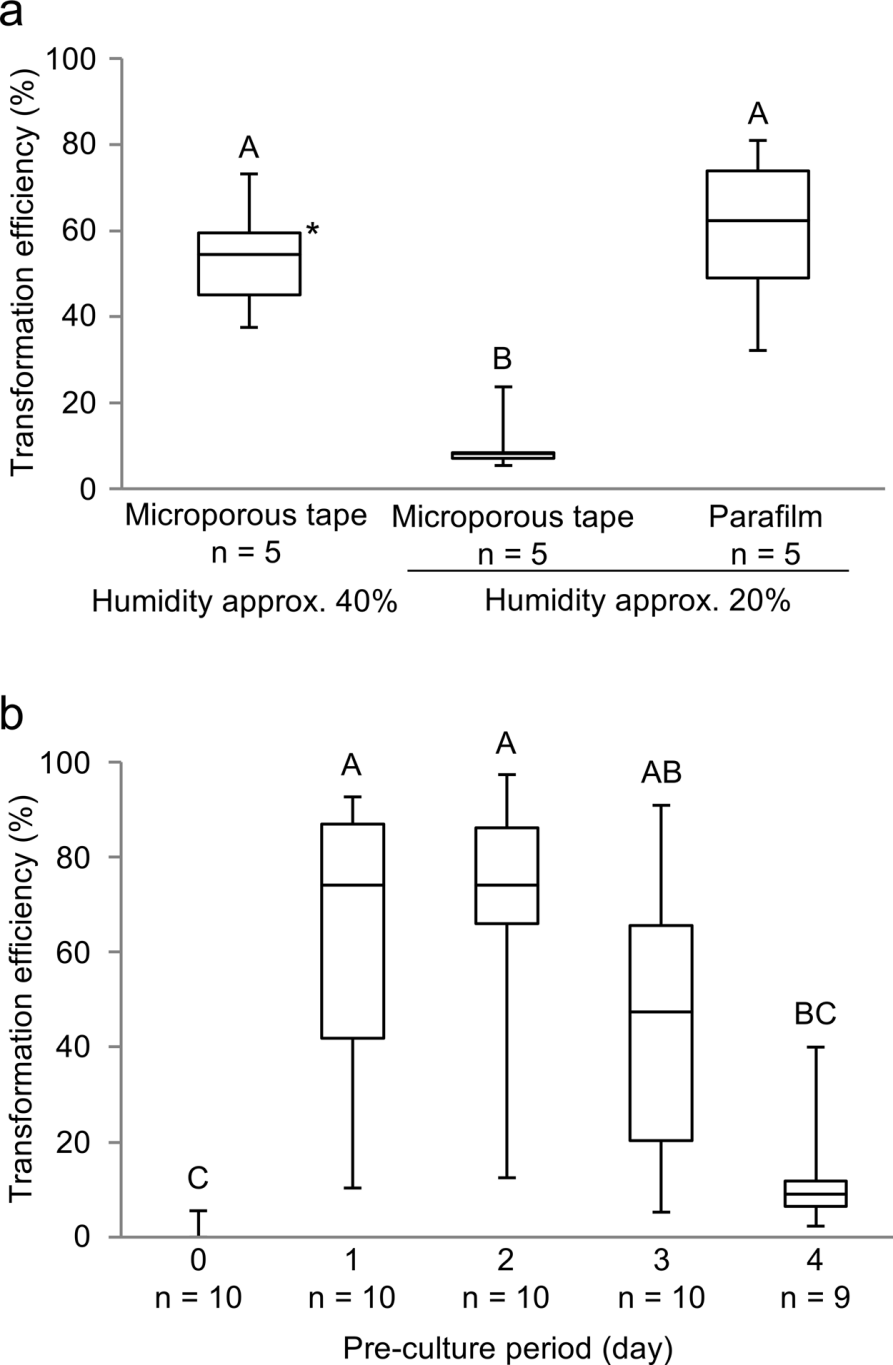
Effect of sealing culture dishes with Parafilm on transformation efficiency. (**a**) Comparison among the use of microporous tape to seal Petri dishes during co-culture in a culture room at approximately 40% and 20% humidity, and the use of Parafilm to seal the Petri dish during co-culture in a culture room at approximately 20% humidity. For both examinations using microporous tape, gemmalings were subjected to a one-day pre-culture before a three-day co-culture. For examinations using Parafilm, gemmalings were subjected to a one-day pre-culture before a two-day co-culture. All examinations were performed with *Agrobacterium* strain GV2260 under light. Different uppercase letters (A, B) indicate a significant difference (Tukey’s test; P < 0.05). *These raw data were reported in Tsuboyama-Tanaka & Kodama 2015. (**b**) Effect of the duration of gemmaling pre-culture prior to the use of Petri dishes sealed with Parafilm during co-culture. All examinations were performed after a two-day co-culture with *Agrobacterium* strain GV2260 under light. Different uppercase letters (A, B, C) indicate a significant difference (Tukey-Kramer’s test; P < 0.05).

In our previous study, gemmalings (BC3-38) were pre-cultured for one day and co-cultured with *Agrobacterium* for three days on ½ B5 medium supplemented with 1% sucrose, which resulted in a median transformation efficiency of 57.0% (mean: 59.2%) (Fig. 2a)^17^. Permeable microporous tape was used to seal the Petri dishes containing the solid medium; therefore, the humidity to which the plants were exposed depended on the humidity of the culture room. The humidity of the culture room was kept at approximately 40% with a humidifier, as in our previous study^17^. In the present study, we tested whether humidity differences in the co-culture step influence transformation efficiency. Without the humidifier, the humidity in the culture room decreased to approximately 20%. When gemmalings were co-cultured with *Agrobacterium* at 20% humidity, although the ½ B5 medium supplemented with 1% sucrose did not dry out completely until observation at 2 weeks after pouring selection buffer, the median transformation efficiency was decreased by 8.1%, and the mean efficiency decreased by 10.5% (Fig. 2a, see also Supplementary Table S1). These results suggested that higher humidities during the co-culture step increase transformation efficiency; however, it can be challenging to control the humidity in culture rooms precisely, because humidity fluctuates depending on the location and/or season.

To maintain a high humidity in the Petri dishes during co-culture, we sealed the dishes with Parafilm, which is more airtight than microporous tape. When using Parafilm, almost all gemmalings co-cultured for three days suffered from an overgrowth of *Agrobacterium* (such as shown in Supplementary Fig. S1), suggesting that the growth of this bacterium is enhanced by high humidity. Since it was difficult to completely eliminate the bacteria in the subsequent selection step when they were overgrown, the co-culture period was shortened to two days when using Parafilm, which increased the median transformation efficiency to 62.3% (mean: 59.6%) in the 20% humidity condition (Fig. 2a, see also Supplementary Table S1). These results indicate that the high humidity in Parafilm-sealed Petri dishes during the co-culture step increases the transformation efficiency, while shortening the required duration of the co-culture period from three days (at 40% humidity when sealed with microporous tape) to two days.

Next, we investigated the pre-culture period of gemmae/gemmalings required when sealing the dishes with Parafilm during the co-culture step. The gemmalings were pre-cultured for 0, 1, 2, 3, and 4 days in a Petri dish sealed with microporous tape, and then co-cultured for two days in a Petri dish sealed with Parafilm, which led to median transformation efficiencies of 0% (mean: 0.6%), 74.1% (mean: 62.6%), 74.1% (mean: 70.3%), 47.4% (mean: 45.8%), and 9.1% (mean: 12.2%), respectively (Fig. 2b, see also Supplementary Table S2). These results indicate that pre-culture periods of one and two days are optimal.

The use of Parafilm-sealed Petri dishes shortened the period required for the AgarTrap co-culture step. For the following investigations, we used fixed conditions of a two-day pre-culture, a two-day co-culture with *Agrobacterium* strain GV2260 in the light in Petri dishes sealed with Parafilm, and no surfactant in the transformation buffer. These conditions were varied as described below, to investigate their impact on transformation efficiency.

### Surfactants in transformation buffer

In previous studies of *Agrobacterium*-mediated transformation, it was reported that the use of surfactants in the co-cultivation medium during co-culture increased the transformation efficiency^20,21^. We therefore examined whether surfactants in the transformation buffer influenced the efficiency of G-AgarTrap.

**Figure 3:**
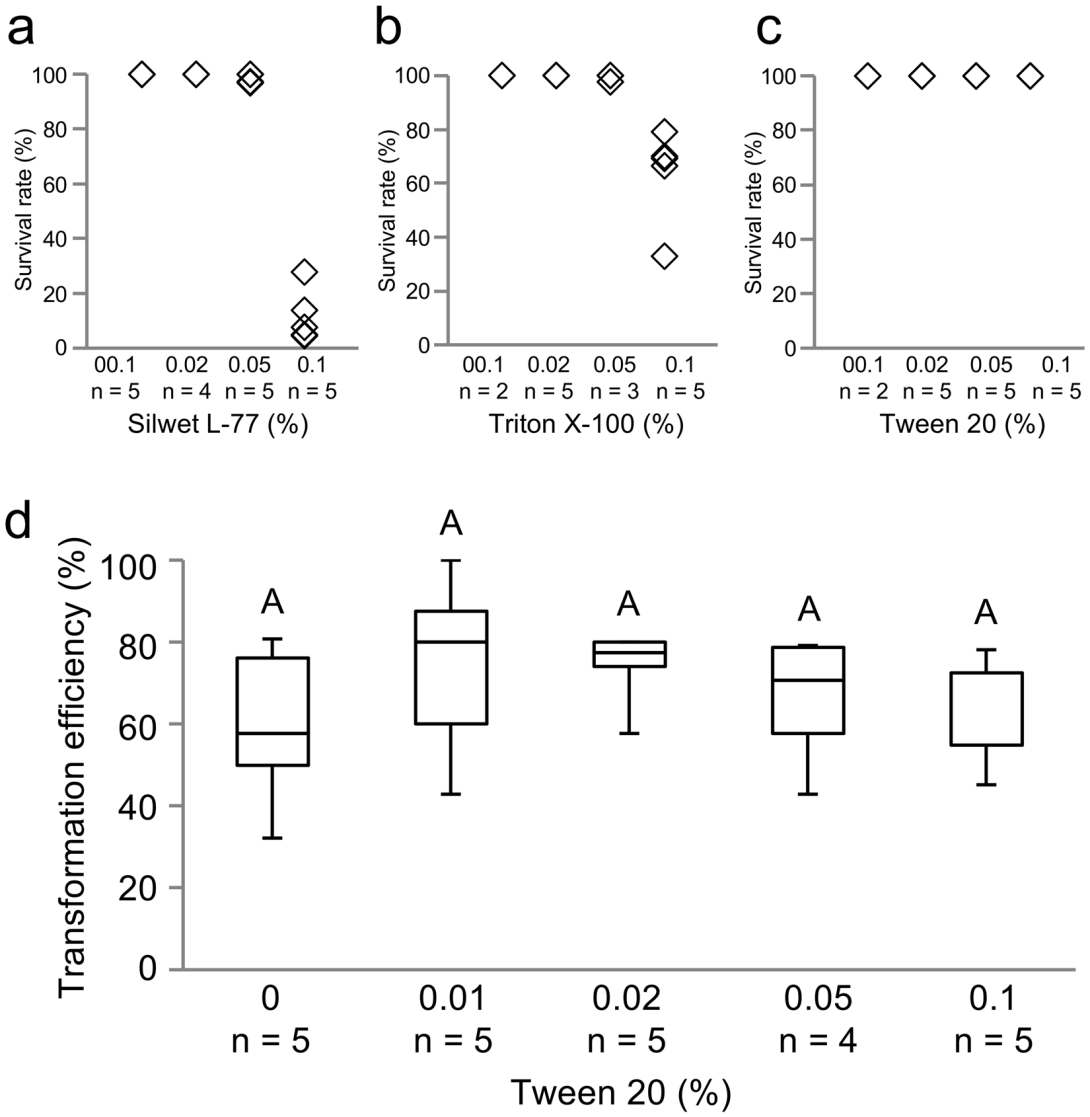
Effect of adding surfactant to the transformation buffer on gemmaling survival rates and transformation efficiency. (**a–c**) The survival rates were estimated for gemmalings treated with various concentrations of Silwet L-77 (**a**), Triton X-100 (**b**), and Tween 20 (**c**). (**d**) The effect of adding Tween 20 to the transformation buffer on transformation efficiency. All examinations were performed following a two-day co-culture with *Agrobacterium* strain GV2260 under light, in Petri dishes sealed with Parafilm. The same uppercase letter (A) indicates no significant difference (Tukey-Kramer’s test; P < 0.05).

To determine a suitable surfactant for *M. polymorpha* transformation, a survival test was performed using three surfactants, Silwet L-77, Triton X-100, and Tween 20. We added various concentrations of these surfactants to the transformation buffer and treated the pre-cultured gemmalings with this buffer. After two days of co-culture, the survival rates of the gemmalings were estimated. Four concentrations of Silwet L-77 (0.01%, 0.02%, 0.05%, and 0.1%) were analyzed, resulting in mean survival rates of 100%, 100%, 98.8%, and 11.7%, respectively (Fig. 3a). When 0.01%, 0.02%, 0.05%, or 0.1% Triton X-100 was used, the mean survival rates of the gemmalings were 100%, 100%, 99.2%, and 63.5%, respectively (Fig. 3b). Because gemmalings could not survive in the higher concentrations of Silwet L-77 and Triton X-100, these surfactants may be toxic to *M. polymorpha*. By contrast, when Tween 20 concentrations of 0.01%, 0.02%, 0.05%, and 0.1% were tested, the mean survival rate was 100% for all concentrations (Fig. 3c). Tween 20 seemed to have no effect on gemmaling growth, and was therefore selected for use as a surfactant.

We assessed whether the use of Tween 20 in the transformation buffer increased the efficiency of G-AgarTrap. Tween 20 concentrations of 0%, 0.01%, 0.02%, 0.05%, and 0.1% resulted in median transformation efficiencies of 57.6% (mean: 59.3%), 80.0% (mean: 74.1%), 77.4% (mean: 73.8%), 70.6% (mean: 65.8%), and 54.8% (mean: 61.1%), respectively (Fig. 3d, see also Supplementary Table S3). These results showed that the use of 0.01–0.02% Tween 20 in the transformation buffer slightly increased the efficiency of G-AgarTrap transformation; however, the differences were not statistically significant. Nevertheless, when the gemmalings were co-cultured in transformation buffer, the solutions lacking surfactant were often repelled by the plants, requiring careful manipulation to ensure proper coverage. When surfactants such as Tween 20 were added to the transformation buffer, this hydrophobicity was counteracted; therefore, the addition of surfactants improves the ease of performing G-AgarTrap transformations.

### *Agrobacterium* strain

**Figure 4:**
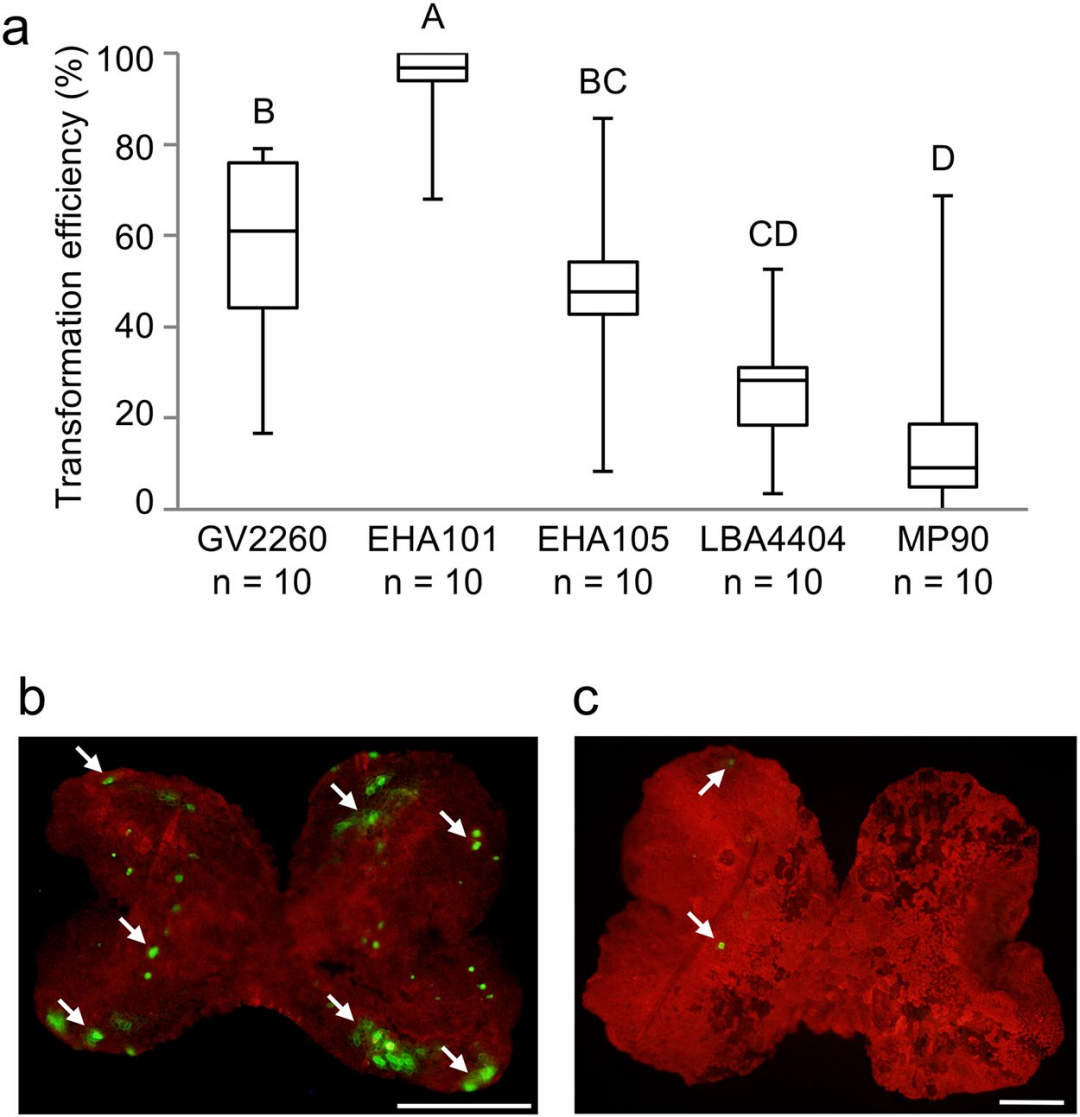
Effect of *Agrobacterium* strain on transformation efficiency. (**a**) The transformation efficiency of G-AgarTrap using five *Agrobacterium* strains, GV2260, EHA101, EHA105, LBA4404, and MP90. All examinations were performed following a two-day co-culture under light, in Petri dishes sealed with Parafilm. Different uppercase letters (A, B, C, D) indicate a significant difference (Tukey’s test; P < 0.05). (**b,c**) Fluorescence images of transient marker expression in a gemmaling transformed using EHA101 (**b**) and MP90 (**c**), cultured for three days after treatment with selection buffer. Red and yellow-green indicate chlorophyll and Citrine fluorescence, respectively. Scale bar, 500 μm. Arrows indicate representative transformed cells.

*Agrobacterium* strains influence the efficiency of *Agrobacterium*-mediated transformations in other plant species, with the most effective strain being dependent on the plant species or transformation method used^22–26^. For the transformation of *M. polymorpha* above, and in the previous G-AgarTrap study, the GV2260 strain was used^17^. To assess the best strain for G-AgarTrap transformation, we compared the efficiencies of the technique using five *Agrobacterium* strains, GV2260, EHA101, EHA105, LBA4404, and MP90^27–31^. The median transformation efficiencies using these strains were 61.0% (mean: 57.6%), 96.7% (mean: 93.8%), 47.6% (mean: 47.2%), 28.3% (mean: 26.2%), and 9.2% (mean: 18.1%), respectively (Fig. 4a, see also Supplementary Table S4). The use of *Agrobacterium* strain EHA101 resulted in over a 90% efficiency in eight out of 10 transformations, and 100% efficiency on four occasions (Fig. 4a, see also Supplementary Table S4). EHA101 was therefore the superior strain for G-AgarTrap with BC3-38, contributing to consistently high levels of transformation efficiency (Fig. 4a, see also Supplementary Table S4), which also resulted in the presence of many transformed cells within each gemmaling (Fig. 4b). Conversely, MP90 was not suitable for G-AgarTrap with BC3-38, as its use resulted in a 0% efficiency for two of 10 transformations, and only ever resulted in one or a few transformed cells within a single gemmaling (Fig. 4a,c, see also Supplementary Table S4).

We assessed the combined use of the most efficient *Agrobacterium* strain, EHA101, and 0.01–0.02% Tween 20 as a surfactant. When gemmalings were transformed with EHA101 in the presence of 0.01% Tween 20, the median transformation efficiency was 95.5% (mean: 93.1%), which was similar to the efficiency of EHA101-mediated transformations without a surfactant (Supplementary Fig. S2, see also Supplementary Table S5). The median transformation efficiency of EHA101 using 0.02% Tween 20 as a surfactant decreased to 17.6% (mean: 40.1%) (Supplementary Fig. S2, see also Supplementary Table S5). Thus, when using EHA101, 0.01% Tween 20 yields better results than 0.02% Tween 20.

### Light/dark condition during co-culture

**Figure 5:**
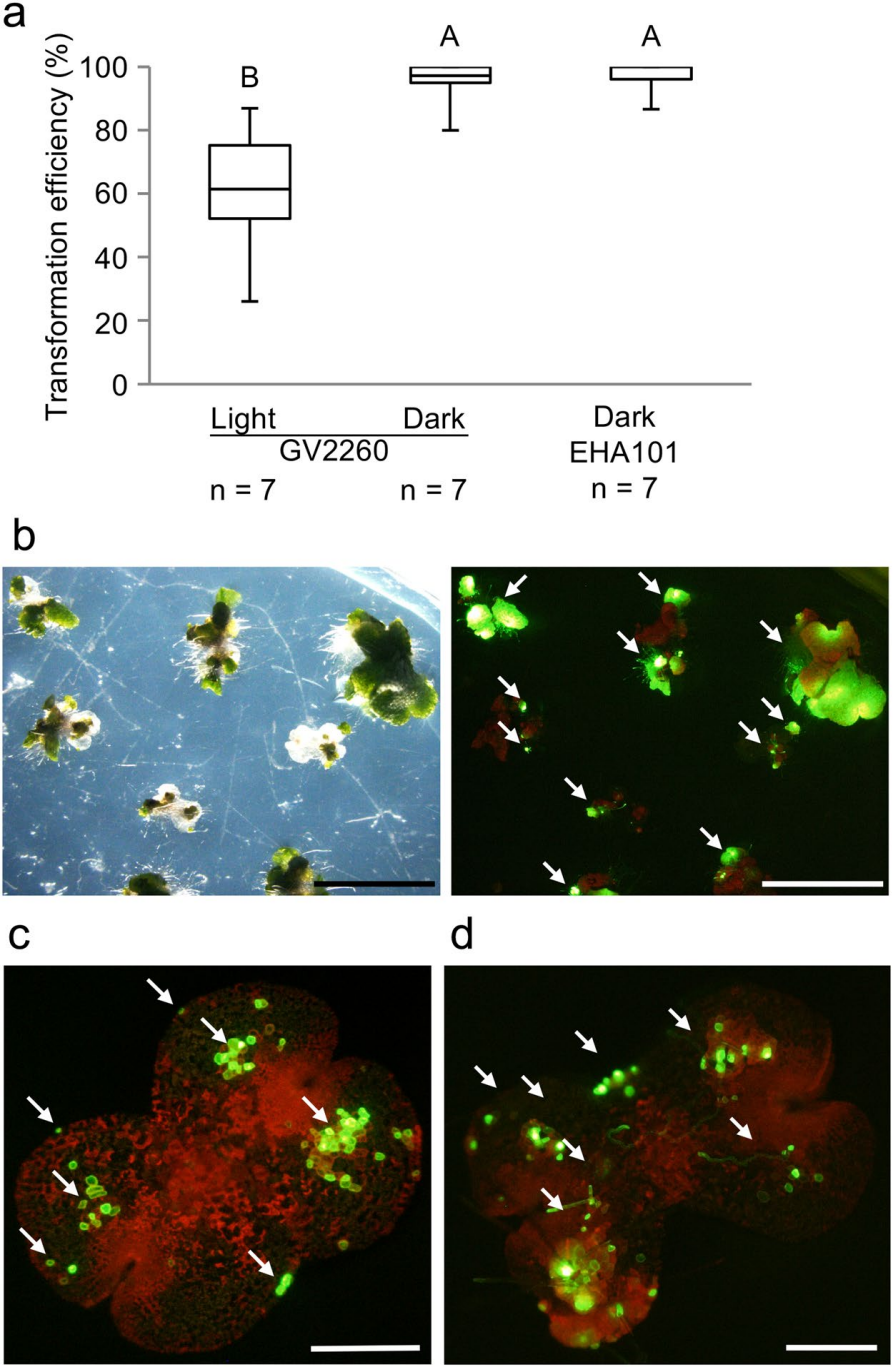
Effect of dark treatment on transformation efficiency. (**a**) Transformation efficiency following co-culture under light and dark conditions using *Agrobacterium* strain GV2260, or following dark culture using strain EHA101. The effects of the light and dark conditions were examined following a two-day co-culture on Petri dishes sealed with Parafilm. Different uppercase letters (A, B) indicate statistically significant differences (Tukey’s test; P < 0.05). (**b**) Transmitted light image (left) and fluorescence image (right) of stable marker expression in transformants generated using EHA101 in the dark, which were cultured under light for two weeks after treatment with selection buffer. Scale bar, 0.5 cm. Arrows indicate representative transformants. (**c**) Fluorescence image of transient marker expression in a gemmaling transformed in darkness using GV2260, and cultured under light for three days after treatment with selection buffer. (**d**) Fluorescence image of transient marker expression in a gemmaling transformed in darkness using EHA101, and cultured in light for five days after treatment with selection buffer. For (**c**,**d**), the scale bar represents 500 μm, red and yellow-green indicate chlorophyll and Citrine fluorescence, respectively, and arrows indicate representative transformed cells.

In previous studies of *Agrobacterium*-mediated transformation, light and dark conditions were reported to influence the transformation efficiency^32–34^. All previous studies of AgarTrap were performed under continuous white light conditions (75 µmol photons m^−2^ s^−1^)^16–18^. When *M. polymorpha* was co-cultured with *Agrobacterium* strain GV2260, the median transformation efficiencies under light and dark conditions were 61.5% (mean: 61.3%) and 97.1% (mean: 95.3%), respectively (Fig. 5a, see also Supplementary Table S6). Additionally, the combined use of the most efficient *Agrobacterium* strain, EHA101, and dark conditions during the co-culture period resulted in a median transformation efficiency of 100% (mean: 97.0%). Of the seven transformations performed in darkness using EHA101, a transformation efficiency of 100% was achieved five times (Fig. 5a,b, see also Supplementary Table S6). Numerous cells in each gemmaling were transformed under the dark condition when using either GV2260 or EHA101 (Fig. 5c,d). Thus, for G-AgarTrap transformation of *M. polymorpha*, the transformation efficiency when gemmalings were co-cultured with *Agrobacterium* under dark conditions was higher than that under light conditions.

### G-AgarTrap using other selectable antibiotics

Four selectable antibiotics, hygromycin (10 mg/L), gentamycin (100 mg/L), chlorsulfuron (0.5 µM), and G418 (5 mg/L), were previously used for *Agrobacterium*-mediated transformation of *M. polymorpha*^35^. Because we optimized G-AgarTrap using only hygromycin (10 mg/L), we evaluated the transformation efficiency of optimized G-AgarTrap using the other three antibiotics: gentamycin, chlorsulfuron, and G418. When we performed optimized G-AgarTrap using hygromycin, gentamicin, chlorsulfuron, and G418 for selection of transformants, their median transformation efficiencies were 100% (mean: 100%), 0.0% (mean: 0.0%), 100% (mean: 98.0%), and 37.9% (mean: 44.0%), respectively (Supplementary Fig. S4). These results indicate that hygromycin and chlorsulfuron can be used as high efficiency selectable antibiotics for optimized G-AgarTrap.

## Discussion

To improve the efficiency of G-AgarTrap transformation of *M. polymorpha*, we optimized the co-culture steps. Among the four factors (humidity, surfactant in the transformation buffer, *Agrobacterium* strain, and light/dark condition) tested, humidity, *Agrobacterium* strain, and light/dark condition could be adjusted to improve transformation efficiency.

Because AgarTrap is performed on solid medium, we predicted that humidity might influence the transformation efficiency. We found that high humidities during co-culture increased transformation efficiency, and that sealing the Petri dishes with Parafilm instead of microporous tape could overcome the problem of low culture room humidity. The high humidity also enhanced *Agrobacterium* growth, suggesting that this bacterium is sensitive to drying out. Sealing the Petri dishes with Parafilm might better maintain a high internal humidity than sealing the Petri dishes with microporous tape. The enhanced *Agrobacterium* growth observed in Petri dishes sealed with Parafilm might promote transformation efficiency; however, the overgrown bacteria were difficult to completely eliminate in the subsequent selection step of G-AgarTrap. When Parafilm was used to seal the Petri dishes during two days of co-culture, efficient pre-culture periods were one and two days. This result was consistent with our previous study using microporous tape-sealed Petri dishes, in which the humidity was approximately 40%^17^. This suggests that the gemmaling cell states arising after 1–2 days of pre-culture might be the most suitable for transformation.

In the *Agrobacterium*-mediated transformation of *Arabidopsis thaliana*, the use of a surfactant, Silwet L-77, increases the transformation efficiency by reducing the surface tension of the aqueous solution^20,36^. In the present study, we did not find any significant improvement in transformation efficiency when using a range of surfactants; however, the addition of surfactants simplified the procedure by reducing the hydrophobicity of the gemmalings, which otherwise repelled the transformation solution. When 0.05% and 0.1% Tween 20 were used, the transformation efficiency using *Agrobacterium* GV2260 was decreased relative to the efficiency when using 0.01% and 0.02% Tween 20 solutions, even though ~1% Tween 20 did not harm the *M. polymorpha* gemmalings. The solutions did not appear to affect the survival rate of *M. polymorpha*; therefore, the higher concentrations (0.05% and 0.1%) of Tween 20 might affect the bacterium itself. The inclusion of Tween 20 when using the more effective EHA101 strain also requires caution, because the transformation efficiency was greatly decreased with a 0.02% concentration of Tween 20 in the transformation solution. EHA101 might therefore be more sensitive to Tween 20 than GV2260.

The transformation efficiency of G-AgarTrap varied significantly with the use of different *Agrobacterium* strains; the strains yielding the highest and lowest efficiencies were EHA101 and MP90, respectively. In a previous study using tomato (*Solanum lycopersicum*), it was suggested that differences in transformation efficiency using different *Agrobacterium* strains was caused by variations in plant tissue mortality^25^, which might also be the case in the present study. Additionally, for many methods using *Agrobacterium*-mediated plant transformation, the co-culture medium was optimized for transformation, but was also used for the culture of both plant material and *Agrobacterium*. By contrast, in AgarTrap, the co-culture was performed on solid medium (½ B5 supplemented with 1% sucrose in agar) optimized for the growth of *M. polymorpha*, but not optimized for *Agrobacterium*. Thus, the solid medium might negatively affect *Agrobacterium*, leading to differences in transformation efficiency as a result of differences in the adaptability of the *Agrobacterium* strains to the medium.

The transformation efficiency of BC3-38 gemmalings was increased by using EHA101 instead of GV2260, which is the *Agrobacterium* strain commonly used in *Marchantia* transformation^8,16–18^. Although the chromosomal backgrounds of both GV2260 and EHA101 were the same as C58^28,29^, their Ti-plasmids were different: pTiB6S3 in GV2260^28^ and pTiBo542 in EHA101^29^. pTiBo542 was reported as a super virulent plasmid that contributes high infectivity and wide host range^37^. Thus, pTiBo542 might be effective in the transformation of BC3-38 gemmalings. EHA105 was developed by removing the kanamycin resistance gene from pTiBo542 in EHA101^30^. Therefore, the EHA101 and EHA105 strains should be genetically almost identical^30^. However, in G-AgarTrap, we found a remarkable difference in transformation efficiency when using EHA101 or EHA105. Thus, these strains might be less genetically similar than previously thought. This possibility remains to be investigated.

Previous reports using intact tobacco (*Nicotiana tabacum*) seedlings, *A. thaliana* root segments, and tepary bean (*Phaseolus acutifolius*) calli suggested that light enhanced transformation efficiency^32,34^, but another report using carnation (*Dianthus caryophyllus*) stem explants reported that dark conditions resulted in a higher proportion of transformants^33^. No significant differences in transformation efficiency were observed between light and dark conditions in garlic (*Allium sativum*)^38^. These conflicting reports suggest that the effects of light on transformation efficiency might depend on the plant species or tissue used. In the present study, we found that performing the co-culture in darkness significantly enhanced the transformation efficiency. The dark-mediated improvement in transformation efficiency for carnation stem explants was previously suggested to be caused by an increased susceptibility to infection in the etiolated tissue, and/or by enhanced *Agrobacterium* activation^33^. A subsequent report confirmed that *Agrobacterium* activation is greater in darkness^39^. Plants are more susceptible to infection by pathogens at night, because the reactive oxygen species produced by photosynthesis enhance their resistance to attack^40^. Taken together, we hypothesize that the dark-mediated activation of *Agrobacterium* and the increased susceptibility to infection in the gemmaling cells in darkness result in the observed improvement in transformation efficiency when performing the AgarTrap co-culture in darkness compared with in light.

Under low humidity conditions (approximately 20%), use of a Petri dish sealed with Parafilm and co-culture in darkness greatly improved the transformation efficiency of G-AgarTrap. Therefore, we also tested Parafilm and darkness in the S- and T-AgarTrap methods. In S-AgarTrap under the low humidity condition (approximately 20%), the transformation efficiencies with microporous tape, Parafilm, and Parafilm with darkness were 3.4% (mean: 7.4%), 10.2% (mean: 11.7%), and 0.0% (mean: 0.0%), respectively (Supplementary Fig. S5). Use of the Petri dish sealed with Parafilm slightly increased the transformation efficiency in S-AgarTrap. By contrast, no transformants were produced in the dark treatment; thus dark treatment negatively affected the transformation efficiency of S-AgarTrap. Under light conditions, the sporelings grew during co-culture with *Agrobacterium*. By contrast, growth of sporelings appeared to stop under dark conditions. Thus, the sporelings that had been pre-cultured for three days might be too immature to be infected by *Agrobacterium* under darkness. In T-AgarTrap under low humidity (approximately 20%), the transformation efficiencies with microporous tape, Parafilm, and Parafilm in darkness were 52.7% (mean: 55.1%), 77.8% (mean: 69.3%), and 75.0% (mean: 80.8%), respectively (Supplementary Fig. S6). Use of the Petri dish sealed with Parafilm slightly increased the transformation efficiency, and dark treatment had little effect on transformation efficiency in T-AgarTrap. Even when the humidity was approximately 20%, the transformation efficiency of T-AgarTrap was moderate (52.7%). Because the pieces of thallus were damaged by cutting, they might be prone to infections by *Agrobacterium*, even under low humidity and light conditions. When the Petri dish was sealed by Parafilm in T-AgarTrap, overgrowth of *Agrobacterium* often occurred (9 out of 12 times in this study). Therefore, we do not recommend sealing the Petri dish with Parafilm in T-AgarTrap.

In a previous study comparing the efficacy of the four antibiotics (hygromycin, gentamicin, chlorsulfuron, and G418) used for *Agrobacterium*-mediated transformation of *M. polymorpha*, the transformation efficiencies were reported to be almost the same when sporelings were used as materials^35^. When chlorsulfuron was used as a selectable antibiotic, it could be used in the improved G-AgarTrap method in addition to hygromycin. The combination of hygromycin and chlorsulfuron should be suitable for double transformation using G-AgarTrap. On the other hand, the transformation efficiency using G418 was much lower than that using hygromycin and chlorsulfuron. No transformants were obtained when using gentamicin; a dose of 100 mg/L gentamicin immediately killed the BC3-38 gemmalings. Thus, the transformed cells of BC3-38 gemmalings might not be able to withstand gentamicin treatment. Similarly, 5 mg/L G418 might be too strong for cells of BC3-38 gemmalings. Therefore, the concentrations of gentamicin and G418 would need to be optimized if these antibiotics were to be used in the improved G-AgarTrap method.

In this study, we successfully developed a highly efficient G-AgarTrap procedure by making several modifications (high humidity, darkness, *Agrobacterium* strain EHA101) to the co-culture step. The improved G-AgarTrap technique will benefit future molecular biology studies of *M. polymorpha*. Furthermore, deciphering the biological mechanisms underpinning the benefits of these improvements may suggest strategies to improve the efficiency of *Agrobacterium*-mediated transformation of various plant species.

## Methods

### Plant materials and growth conditions

*Marchantia polymorpha* (L.) gemmae/gemmalings of BC3-38, the female line of the third backcross generation created in the crossing of Takaragaike-1 (Tak-1; male line) and Takaragaike-2 (Tak-2; female line), were used in this study. BC3-38 was provided by Dr. Takayuki Kohchi (Kyoto University, Kyoto, Japan). The plants were maintained on half-strength Gamborg’s B5 (½ B5) medium^41,42^ containing 1% agar (BOP; SSK Sales Co., Ltd., Shizuoka, Japan), pH 5.5, in a 90-mm disposable sterile Petri dish. *M. polymorpha* tissues were illuminated with 75 µmol photons m^−2^ s^−1^ continuous white light (FL40SW; NEC Corporation, Tokyo, Japan) in a culture room maintained at around 22 °C with air conditioning and approximately 20% humidity without humidifier. The gemmae/gemmalings subjected to G-AgarTrap transformation were obtained from one- to two-month-old thalli.

### G-AgarTrap

The basic procedure and protocol of G-AgarTrap were previously reported^16,43^. Gemmae were sown on approximately 10 mL ½ B5 solid medium (1% agar) supplemented with 1% sucrose, pH 5.5, in a 60-mm disposable sterile Petri dish, and pre-cultured for 0–4 days. For the co-culture, 1 mL transformation buffer (10 mM MgCl_2_; 10 mM MES-NaOH, pH 5.7; 150 µM acetosyringone; *Agrobacterium* OD_600_ = 0.5) was poured over the gemmalings, with the excess being removed after 1 min using an aspirator or micropipette to avoid overgrowth of *Agrobacterium*. Four factors were considered, including sealing of the Petri dish with Parafilm, the *Agrobacterium* strain used, the addition of a surfactant (0.01–0.1% Tween 20) in the transformation buffer, and dark treatment during the 2 days co-culture period. After co-cultivation, the *Agrobacterium* was twice washed from the gemmalings and solid medium with 4 mL sterile water, and then 1 mL selection buffer containing antibiotics (100 μg hygromycin B and 1 mg Claforan) was poured over the gemmalings and the solid medium. After culturing for a few weeks, the transformed cells had grown and the non-transgenic cells had died^16^. For use in further applications, the transformants were genetically purified, because it is possible that the transformants may consist of several independently transformed cells. The AgarTrap procedure and operations after AgarTrap were described in Tsuboyama and Kodama 2018^43^.

### *Agrobacterium* preparation for G-AgarTrap

*Agrobacterium tumefaciens* harboring the *pMpGWB103-Citrine* vector, which encodes bacterial aminoglycoside resistance (aadA), was stored in 30% glycerol at −80 °C. On the same day that the gemmae were sown on ½ B5 medium (the first step in the G-AgarTrap procedure), *Agrobacterium* was streaked on Luria-Bertani (LB) solid medium (1.5% agar) supplemented with 100 mg L^–1^ spectinomycin and incubated at 28 °C for 2–3 days (Supplementary Fig. S1a). The *Agrobacterium* was then suspended in transformation buffer at OD_600_ = 0.5 (Supplementary Fig. S1b). Surfactant (0.01–0.1% Silwet L-77, Triton X-100, or Tween 20) was included in the transformation buffer. A 1-mL aliquot of transformation buffer was poured onto each Petri dish during the co-culture step.

### Microscopy observation

*M. polymorpha* gemmalings were observed using a MZ16F stereo fluorescence microscope (Leica Microsystems, Wetzlar, Germany). Chlorophyll fluorescence and Citrine fluorescence (in transgenic cells) were determined using a fluorescence module (excitation filter: 480/40 nm; barrier filter: LP 510 nm). Images were taken using a DP73 digital camera (Olympus, Tokyo, Japan).

### Transformation efficiency

The transformation efficiency was evaluated using the binary vector *pMpGWB103-Citrine*, which was transformed into *Agrobacterium* as described previously^16–18^. The T-DNA of *pMpGWB103-Citrine* possessed two marker genes encoding hygromycin B phosphotransferase and Citrine fluorescent protein^16–18^. To identify stable transformants, *M. polymorpha* gemmalings were selected for their ability to grow on the antibiotic hygromycin B (10 μg mL^−1^), and their yellow fluorescence was observed using fluorescence microscopy more than two weeks after the selection buffer was poured (transient expression of Citrine has not been observed after this time)^16–18^. A gemmaling containing one or more transformed cells was considered transformed^17^. The transformation efficiency (%) was calculated as the number of transformed gemmalings divided by the total number of gemmalings, multiplied by 100. Approximately 10–80 gemmalings per Petri dish were used per transformation. The median transformation efficiency was considered to be representative, and the mean was also reported to facilitate comparisons with previous studies. Statistics were analyzed by t-test, Tukey’s test, or Tukey-Kramer’s test.

## Electronic supplementary material

Supplementary figures and tables
