# Supplementary material for: Improved G-AgarTrap: A highly efficient transformation method for intact gemmalings of the liverwort Marchantia polymorpha
Source: Sci Rep. 2018 Jul 17;8:10800. doi: 10.1038/s41598-018-28947-0 (PMC6050340; doi:10.1038/s41598-018-28947-0)
Supplement: Supplementary file 1 — Supplementary figures and tables [file 41598_2018_28947_MOESM1_ESM.pdf]

Supplementary Figures and Tables

Improved G-AgarTrap: A highly efficient transformation method for intact gemmalings of the liverwort *Marchantia polymorpha*

Shoko Tsuboyama[1], Satoko Nonaka[2], Hiroshi Ezura[2], and Yutaka Kodama[1*]

[1]Center for Bioscience Research and Education, Utsunomiya University, Tochigi 321-8505, Japan

[2]Tsukuba Plant Innovation Research Center, University of Tsukuba, Ibaraki 305-8572, Japan

* Corresponding Author: Yutaka Kodama

E-mail: kodama@cc.utsunomiya-u.ac.jp

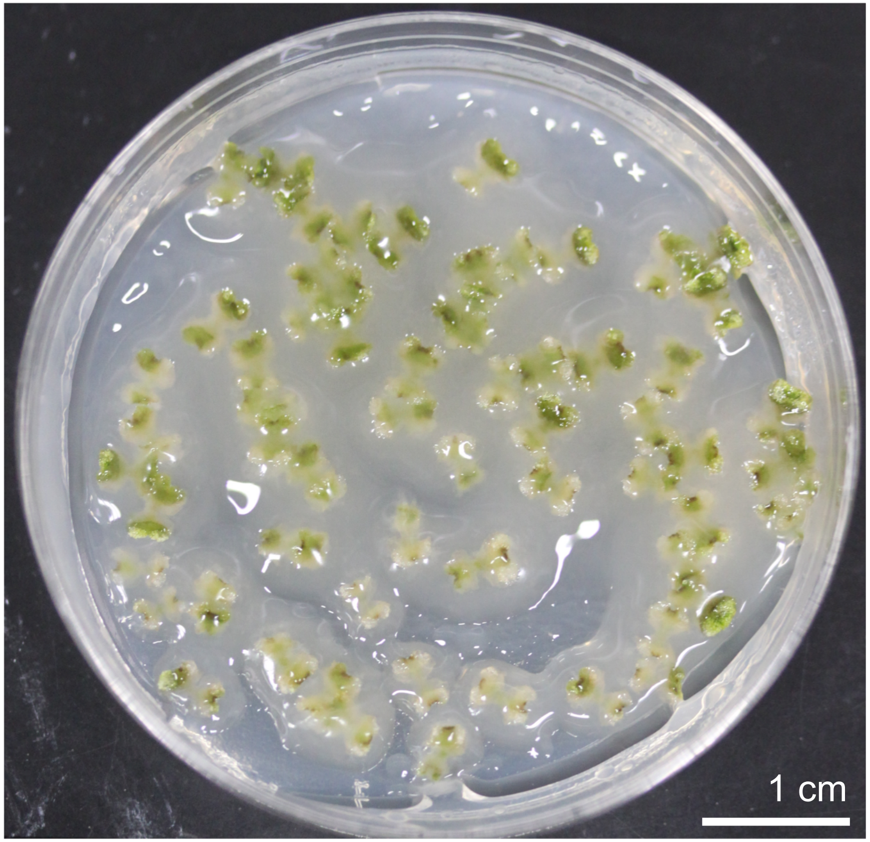

Fig. S1 *Agrobacterium* overgrowth. This photograph was taken 7 days after selection. *Agrobacterium* coated the *M. polymorpha* gemmalings. We were unable to remove overgrown *Agrobacterium* though washing and selection.

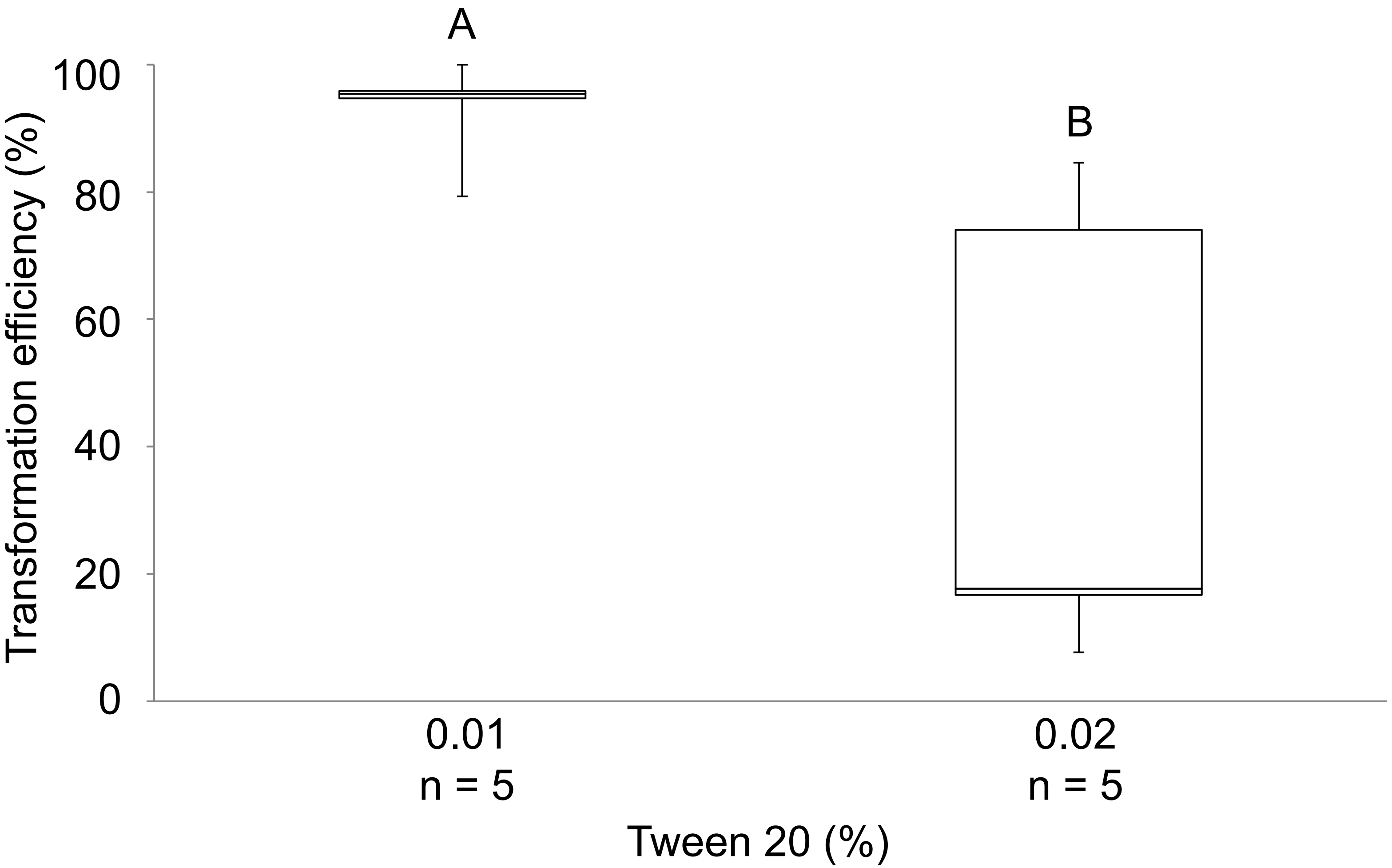

Fig. S2 Transformation efficiency of EHA101 using Tween 20 as a surfactant. The median transformation efficiency of EHA101 with 0.01% Tween 20 was 95.5%, and that with 0.02% Tween 20 was 17.6%. There was a significant difference between transformation efficiency with 0.01% and 0.02% Tween 20 (T test, $p>0.05$).

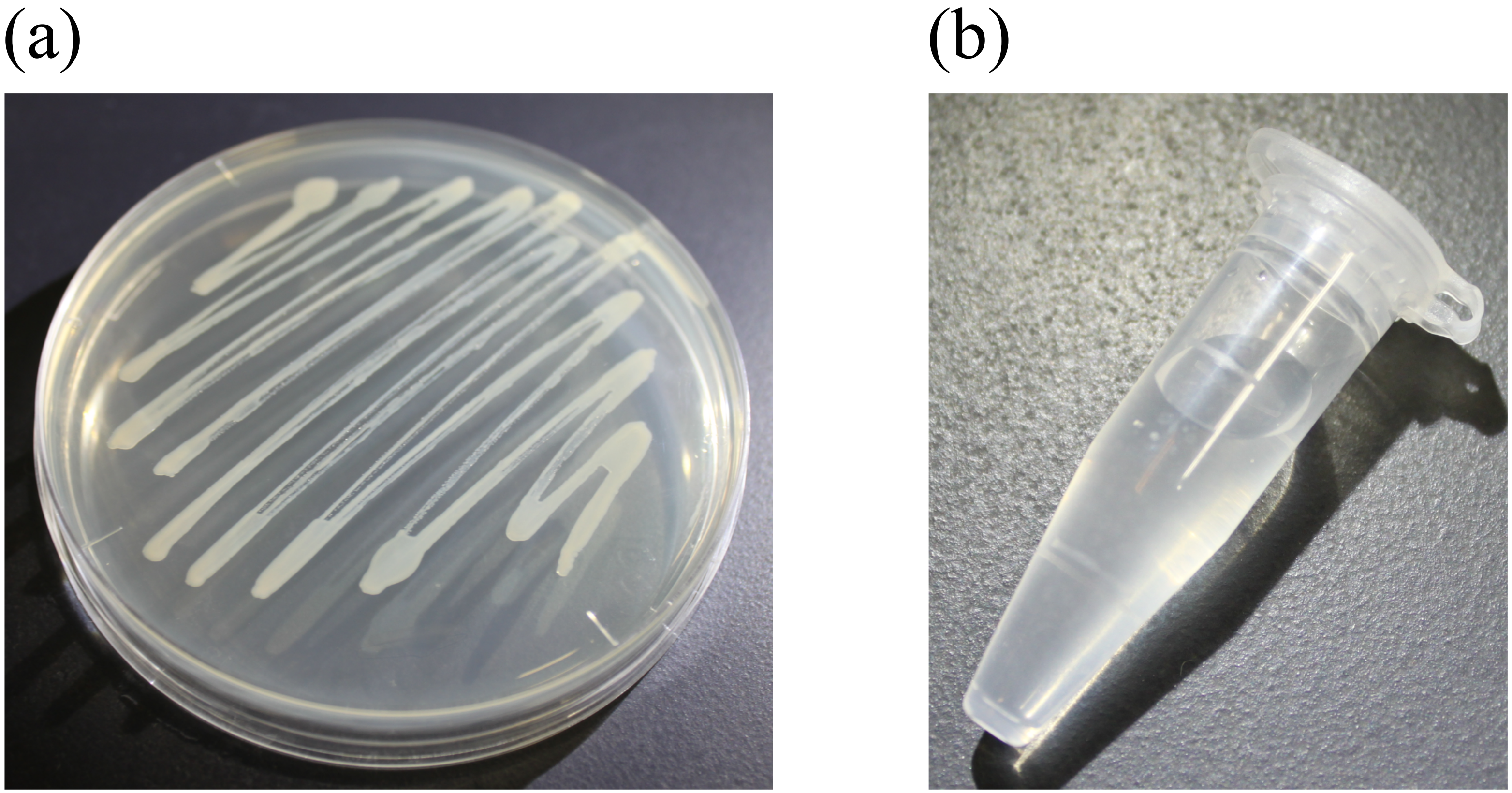

Fig. S3 caption follows the two-panel figure.

Fig. S3 Preparation of *Agrobacterium*. (a) *Agrobacterium* streaked on Luria–Bertani (LB) solid medium, and cultured for 2 days at 28˚C. (b) *Agrobacterium* suspended in 1 mL of transformation buffer at $OD_{600} = 0.5$.

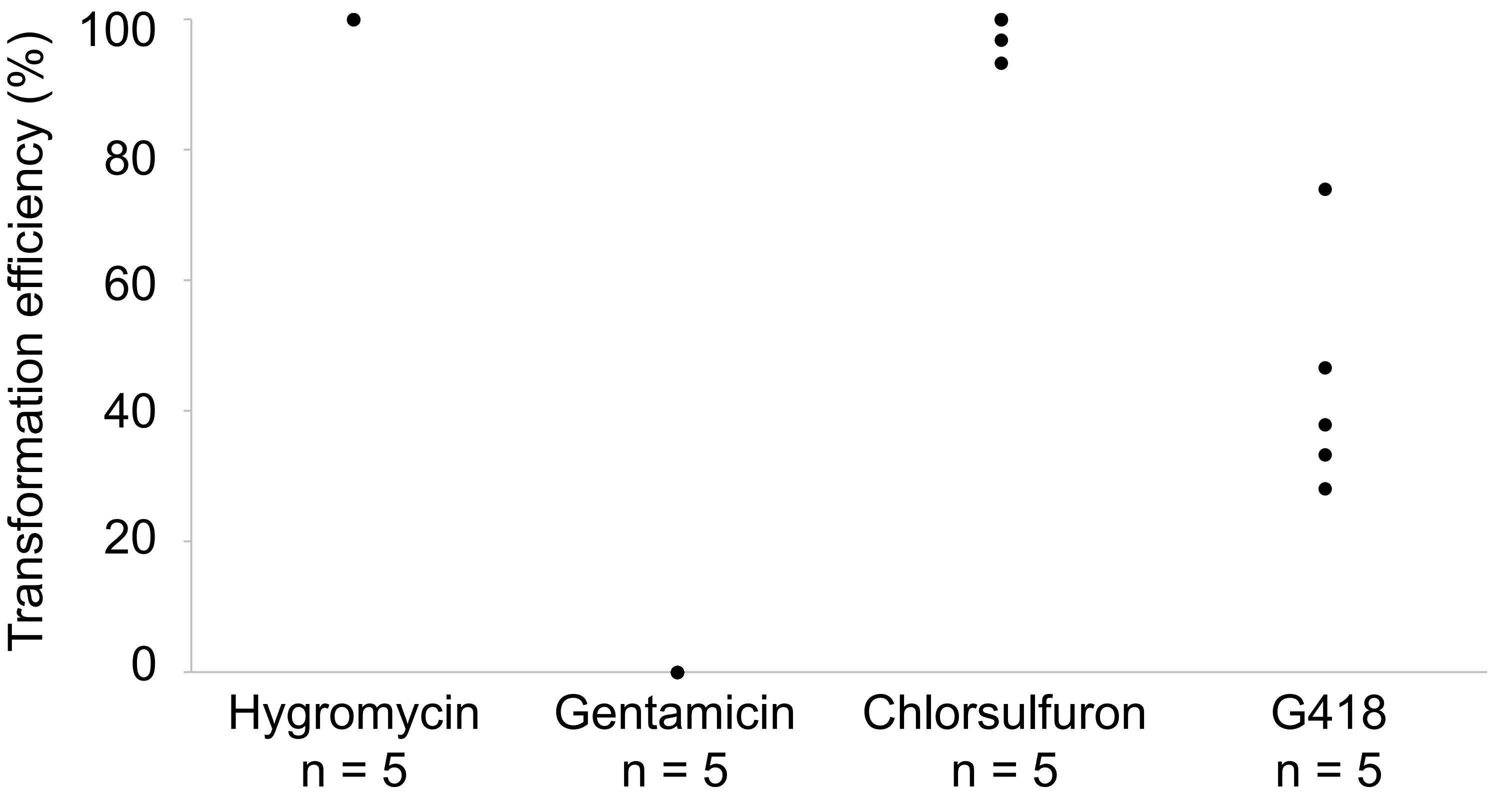

Fig. S4 Transformation efficiency of G-AgarTrap using hygromycin (10 mg/L), gentamicin (100 mg/L), chlorsulfuron (0.5 μM), and G418 (5 mg/L) for selection of transformants. All examinations were performed with 3 days pre-culture and 2 days co-culture (Parafilm and darkness). The gemmalings were transformed by *Agrobacterium* strain EHA101 harboring a binary vector, which is *pMpGWB103-Citrine*, *pMpGWB203-Citrine*, *pMpGWB303-Citrine*, or *pMpGWB403-Citrine* encoding hygromycin phosphotransferase (HPT), gentamicin 3'- acetyltransferase (aacC1), mutated acetolactate synthase (mALS), or neomycin phosphotransferase II (nptII), respectively. The resulting transformants were observed at 2 weeks after pouring selection buffer.

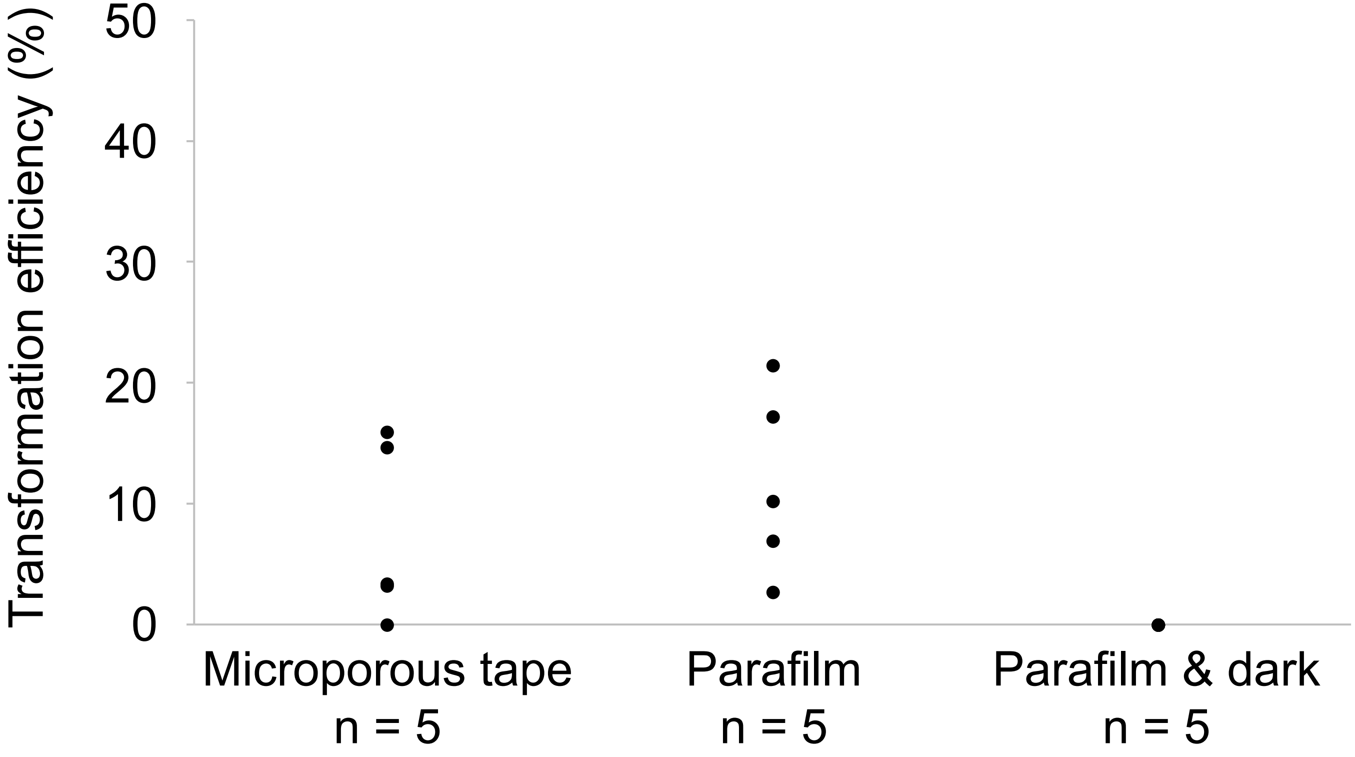

Fig. S5 Effect of Parafilm sealing and dark treatment for S-AgarTrap. The examinations were performed with 3 days pre-culture, 3 days (microporous tape or Parafilm) or 2 days co-culture (Parafilm and darkness), and *Agrobacterium* strain GV2260 harboring *pMpGWB103-Citrine*. The resulting transformants were observed at 2 weeks after pouring selection buffer.

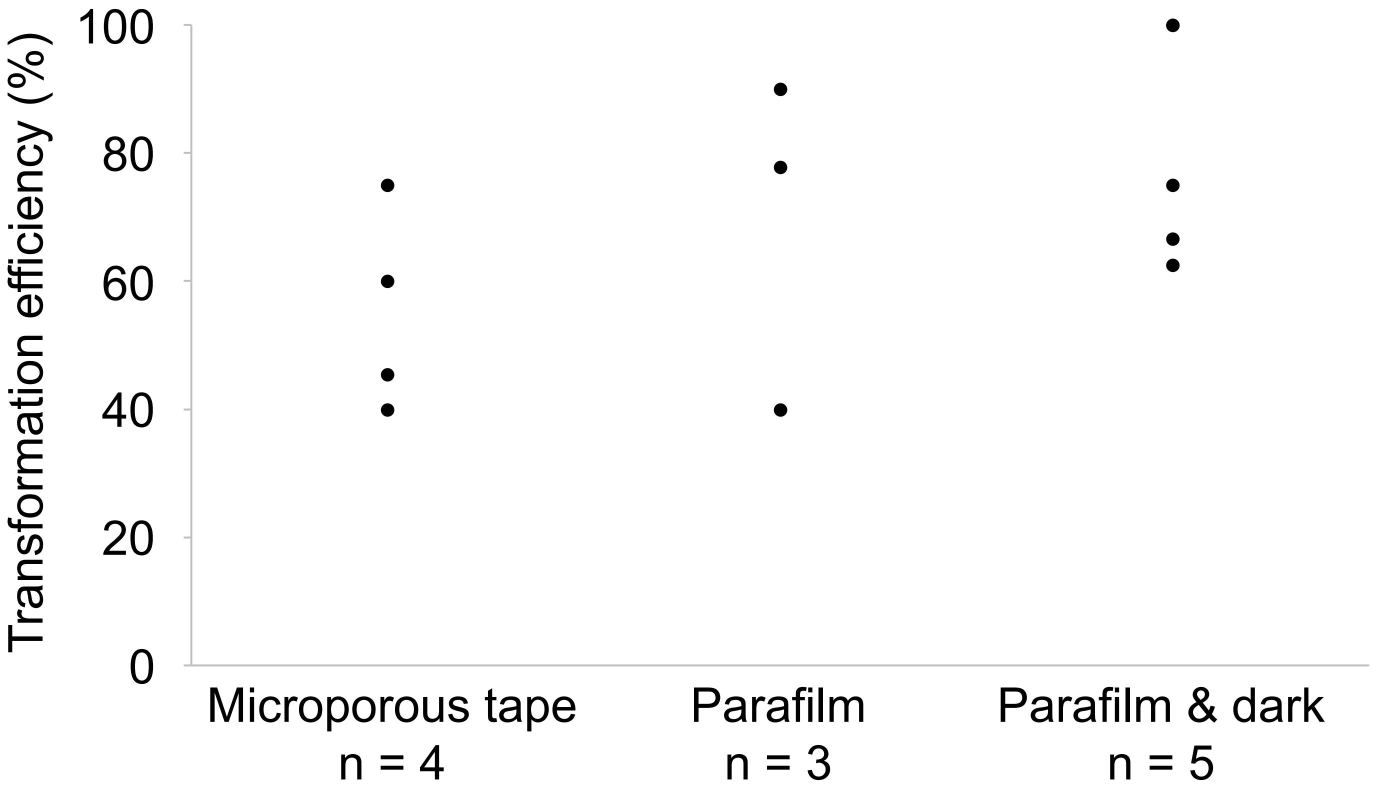

Fig. S6 Effect of Parafilm sealing and dark treatment for T-AgarTrap. All examinations were performed with 0 day pre-culture, 2 days co-culture, and *Agrobacterium* strain GV2260 harboring *pMpGWB103-Citrine*. The resulting transformants were observed at 2 weeks after pouring selection buffer.

Table S1. Effect of humidity in the culture room on transformation efficiency.

Humidity (%)	Total number of gemmalings	Number of transformed gemmalings[a]	Transformation efficiency (%)[b]	Average (%)	SD
20	74	6	8.1	10.5	7.4
	37	2	5.4		
	38	9	23.7		
	28	2	7.1		
	24	2	8.3		
40	42	31	73.8	59.6	19.6
	21	17	81.0		
	53	33	62.3		
	45	22	48.9		
	28	9	32.1		

Actual number of all sown gemmalings and transformed gemmalings.

[a]All transformed gemmalings, based on hygromycin resistance and Citrine fluorescence, were observed by fluorescence microscopy over 2 weeks after pouring selection buffer in order to eliminate transiently expressed cells.

[b]Transformation efficiency was calculated as (number of transformed gemmalings) / (number of all sown gemmalings) x 100, and rounded off to one decimal place.

Table S2 Effect of pre-culture period of gemmae/gemmalings on transformation efficiency.

Pre-culture period (day)	Total number of gemmalings	Number of transformed gemmalings[a]	Transformation efficiency (%)[b]	Average (%)	SD
0	21	0	0	0.6	1.8
	6	0	0		
	17	0	0		
	19	0	0		
	18	1	5.6		
	13	0	0		
	14	0	0		
	13	0	0		
	13	0	0		
	10	0	0		
1	41	38	92.7	62.6	30.6
	53	38	71.7		
	35	27	77.1		
	33	30	90.9		
	31	28	90.3		
	27	5	18.5		
	17	13	76.5		
	19	12	63.2		
	23	8	34.8		
	29	3	10.3		
2	24	3	12.5	70.3	25.4
	15	7	46.7		
	38	37	97.4		
	17	14	82.4		
	25	17	68.0		
	22	17	77.3		
	22	21	95.5		
	16	14	87.5		
	24	17	70.8		

Pre-culture period (day)	Number of all gemmalings	Number of transformed gemmalings[a]	Transformation efficiency (%)[b]	Average (%)	SD
2	26	17	65.4		
3	20	17	85.0	45.8	30.1
	29	13	44.8		
	34	17	50.0		
	24	17	70.8		
	18	9	50.0		
	19	1	5.3		
	13	2	15.4		
	22	20	90.9		
	46	16	34.8		
	46	5	10.9		
4	33	7	21.2	12.2	11.8
	20	8	40.0		
	17	2	11.8		
	22	2	9.1		
	32	1	3.1		
	46	3	6.5		
	21	2	9.5		
	46	3	6.5		
	43	1	2.3		

Actual number of all sown gemmalings and transformed gemmalings.

[a]All transformed gemmalings, based on hygromycin resistance and Citrine fluorescence, were observed by fluorescence microscopy over 2 weeks after pouring selection buffer in order to eliminate transiently expressed cells.

[b]Transformation efficiency was calculated as (number of transformed gemmalings) / (number of all sown gemmalings) x 100, and rounded off to one decimal place.

Table S3 Effect of adding a surfactant, Tween 20, into the transformation buffer on transformation efficiency.

Tween 20 (%)	Total number of gemmalings	Number of transformed gemmalings[a]	Transformation efficiency (%)[b]	Average (%)	SD
0	28	9	32.1	59.3	19.8
	44	22	50.0		
	25	19	76.0		
	33	19	57.6		
	26	21	80.8		
0.01	20	16	80.0	74.1	22.7
	24	21	87.5		
	16	16	100.0		
	28	12	42.9		
	25	15	60.0		
0.02	20	16	80.0	73.8	9.3
	31	24	77.4		
	26	15	57.7		
	40	32	80.0		
	23	17	73.9		
0.05	83	52	62.7	65.8	17.1
	28	22	78.6		
	28	12	42.9		
	48	38	79.2		
0.1	31	17	54.8	61.1	13.7
	29	21	72.4		
	31	14	45.2		
	32	25	78.1		
	31	17	54.8		

Actual number of all sown gemmalings and transformed gemmalings.

[a]All transformed gemmalings, based on hygromycin resistance and Citrine fluorescence, were observed by fluorescence microscopy over 2 weeks after pouring selection buffer in order to eliminate transiently expressed cells.

[b]Transformation efficiency was calculated as (number of transformed gemmalings) / (number of all sown gemmalings) x 100, and rounded off to one decimal place.

Table S4 Effect of *Agrobacterium* strain on transformation efficiency.

Agrobacterium strain	Total number of gemmalings	Number of transformed gemmalings[a]	Transformation efficiency (%)[b]	Average (%)	SD
GV2260	38	30	78.9	57.6	20.7
	31	21	67.7		
	43	34	79.1		
	33	23	69.7		
	27	13	48.1		
	22	9	40.9		
	30	5	16.7		
	35	19	54.3		
	28	12	42.9		
	32	25	78.1		
EHA101	33	33	100.0	93.8	9.9
	31	31	100.0		
	29	29	100.0		
	26	25	96.2		
	32	31	96.9		
	29	28	96.6		
	30	28	93.3		
	25	17	68.0		
	39	34	87.2		
	21	21	100.0		
EHA105	43	20	46.5	47.2	21.0
	29	16	55.2		
	48	4	8.3		
	36	15	41.7		
	34	22	64.7		
	21	18	85.7		
	37	18	48.6		
	35	18	51.4		
	24	11	45.8		

Agrobacterium strain	Number of all gemmalings	Number of transformed gemmalings[a]	Transformation efficiency (%)[b]	Average (%)	SD
EHA105	21	5	23.8		
LBA4404	22	7	31.8	26.2	13.3
	25	7	28.0		
	28	7	25.0		
	43	7	16.3		
	37	5	13.5		
	35	10	28.6		
	38	13	34.2		
	19	10	52.6		
	21	6	28.6		
	29	1	3.4		
MP90	37	8	21.6	18.1	23.0
	32	22	68.8		
	45	4	8.9		
	26	13	50.0		
	29	3	10.3		
	26	2	7.7		
	26	1	3.8		
	21	2	9.5		
	47	0	0.0		
	42	0	0.0		

Actual number of all sown gemmalings and transformed gemmalings.

[a]All transformed gemmalings, based on hygromycin resistance and Citrine fluorescence, were observed by fluorescence microscopy over 2 weeks after pouring selection buffer in order to eliminate transiently expressed cells.

[b]Transformation efficiency was calculated as (number of transformed gemmalings) / (number of all sown gemmalings) x 100, and rounded off to one decimal place.

Table S5 Effect of adding Tween 20 to transformation buffer when using the EHA101 strain on transformation efficiency.

Tween 20 (%)	Total number of gemmalings	Number of transformed gemmalings[a]	Transformation efficiency (%)[b]	Average (%)	SD
0.01	24	23	95.8	93.1	8.0
	22	21	95.5		
	33	33	100.0		
	38	36	94.7		
	29	23	79.3		
0.02	26	2	7.7	40.1	36.2
	34	6	17.6		
	26	22	84.6		
	18	3	16.7		
	27	20	74.1		

Actual number of all sown gemmalings and transformed gemmalings.

[a]All transformed gemmalings, based on hygromycin resistance and Citrine fluorescence, were observed by fluorescence microscopy over 2 weeks after pouring selection buffer in order to eliminate transiently expressed cells.

[b]Transformation efficiency was calculated as (number of transformed gemmalings) / (number of all sown gemmalings) x 100, and rounded off to one decimal place.

Table S6 Effect of dark treatment on transformation efficiency.

Agrobacterium strain	Light condition	Total number of gemmalings	Number of transformed gemmalings[a]	Transformation efficiency (%)[b]	Average (%)	SD
GV2260	Light	23	16	69.6	61.3	20.5
		39	24	61.5		
		26	21	80.8		
		24	13	54.2		
		22	11	50.0		
		23	20	87.0		
		23	6	26.1		
GV2260	Dark	20	16	80.0	95.3	7.1
		20	19	95.0		
		39	37	94.9		
		42	42	100.0		
		42	42	100.0		
		35	34	97.1		
		22	22	100.0		
EHA101	Dark	15	13	86.7	97.0	5.4
		36	36	100.0		
		20	20	100.0		
		19	19	100.0		
		26	24	92.3		
		23	23	100.0		
		12	12	100.0		

Actual number of all sown gemmalings and transformed gemmalings.

[a]All transformed gemmalings, based on hygromycin resistance and Citrine fluorescence, were observed by fluorescence microscopy over 2 weeks after pouring selection buffer in order to eliminate transiently expressed cells.

[b]Transformation efficiency was calculated as (number of transformed gemmalings) / (number of all sown gemmalings) x 100, and rounded off to one decimal place.
